# Supplementary material for: Cell‐type‐specific gene expression and regulation in the cerebral cortex and kidney of atypical Setbp1 S858R Schinzel Giedion Syndrome mice
Source: J Cell Mol Med. 2023 Oct 23;27(22):3565–77. doi: 10.1111/jcmm.18001 (PMC10660642; doi:10.1111/jcmm.18001)
Supplement: Supplementary file 1 — Appendix S1 [file JCMM-27-3565-s002.zip › jcmm18001-sup-0001-v3_Whitlock_SUPP_figures & tables_etal_CellularMolecularMedicine_.docx]

**Table S1: Sample Sheet**

| **sample_ID** | **tissue** | **model/condition** | **age (weeks)** | **sex** |
| --- | --- | --- | --- | --- |
| J1 | right cerebral cortex | S858R | 6 | M |
| J2 | right cerebral cortex | C57BL6/J control | 6 | M |
| J3 | right cerebral cortex | C57BL6/J control | 6 | M |
| J4 | right cerebral cortex | C57BL6/J control | 6 | M |
| J13 | right cerebral cortex | S858R | 6 | M |
| J15 | right cerebral cortex | S858R | 6 | M |
| K1 | right kidney | C57BL6/J control | 6 | M |
| K2 | right kidney | S858R | 6 | M |
| K3 | right kidney | C57BL6/J control | 6 | M |
| K4 | right kidney | S858R | 6 | M |
| K5 | right kidney | C57BL6/J control | 6 | M |
| K6 | right kidney | S858R | 6 | M |

**Figure S1: S858R Variant confirmation in IGV**


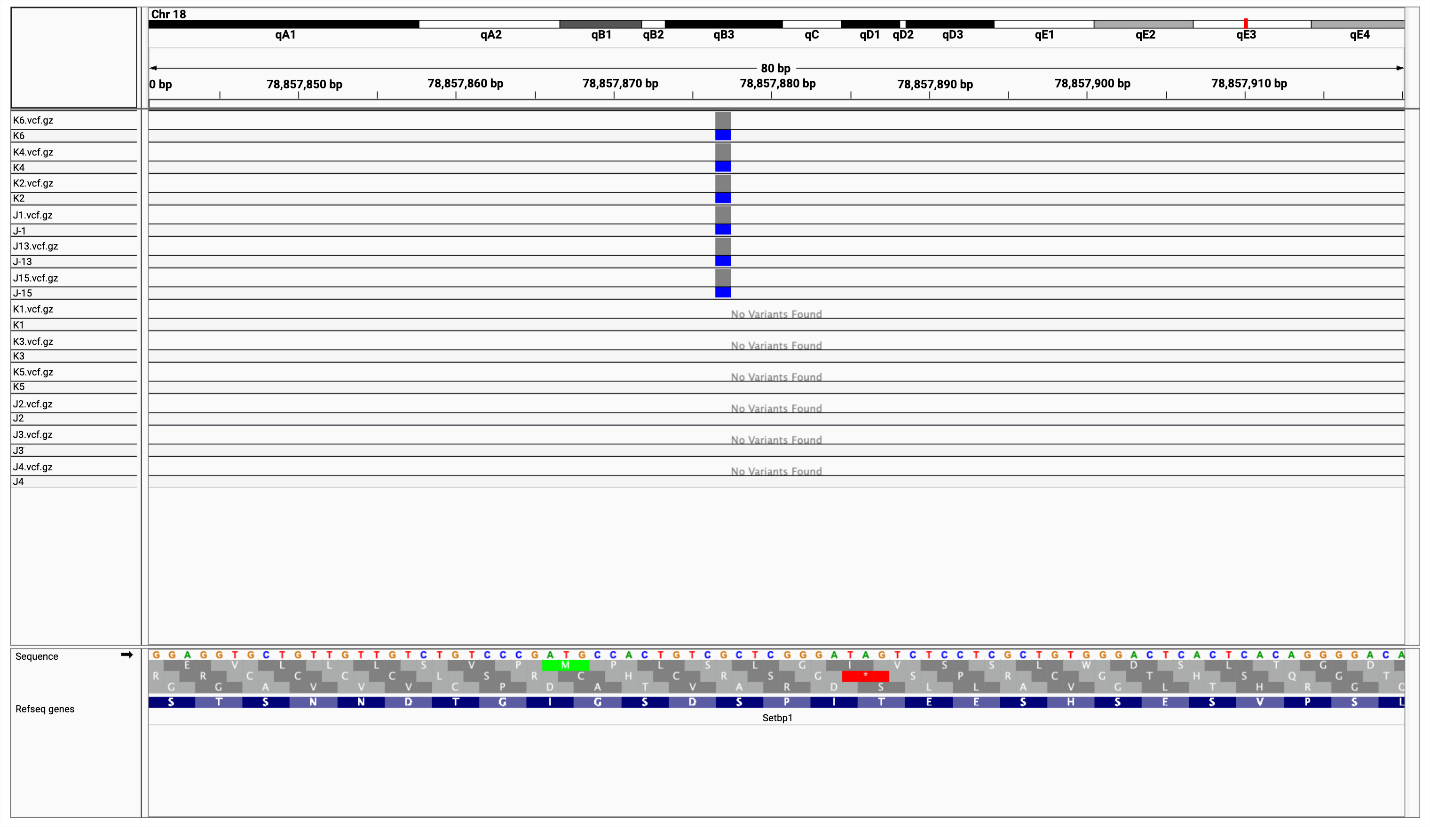


**Figure S2: Setbp1 total protein is significantly increased in S858R compared to WT**


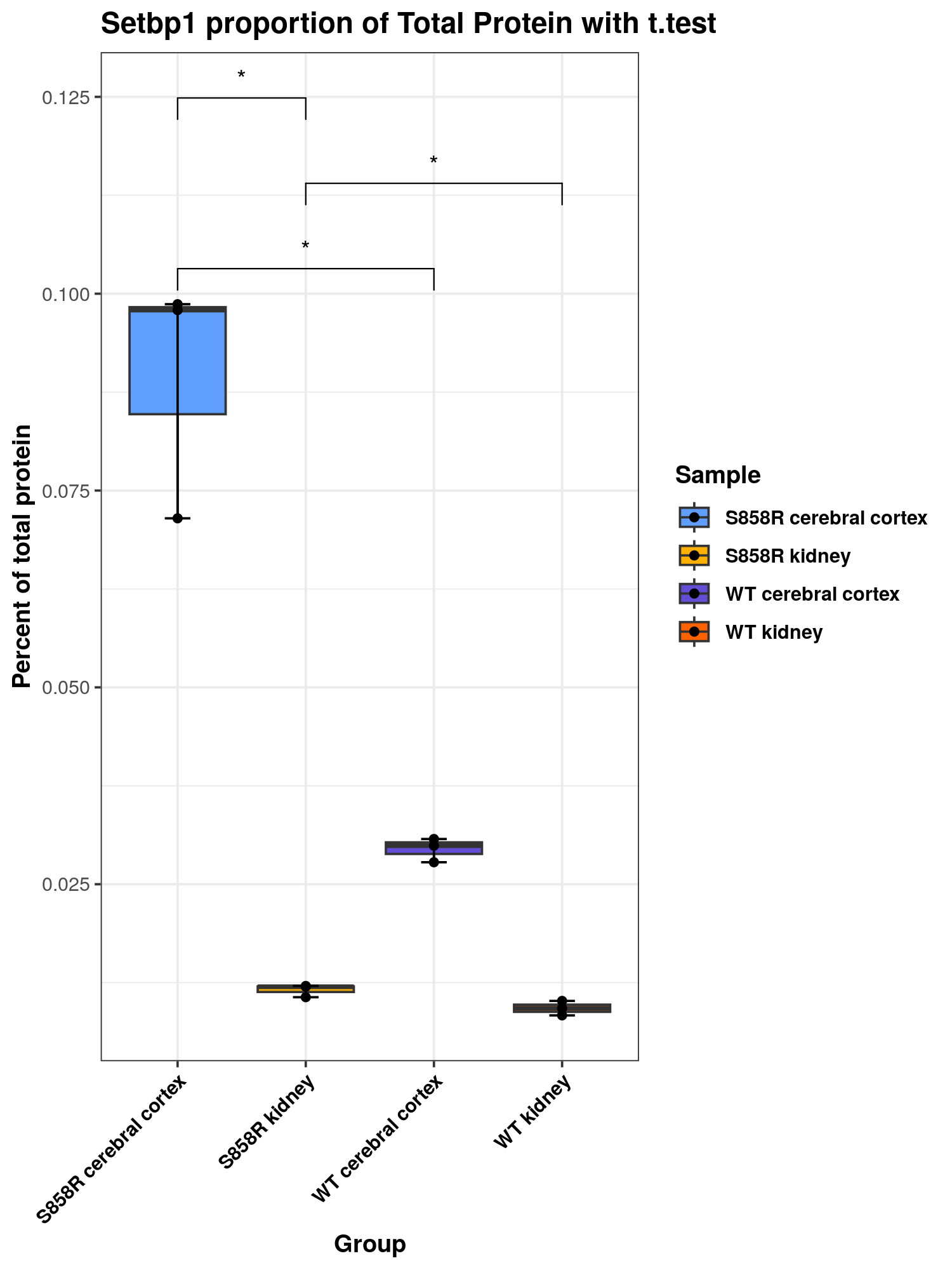


**Table S2: Table of marker genes for cortex and kidney cell types used in cell type annotation**

| **Cerebral Cortex Cell Type** | **Marker Gene(s)** |
| --- | --- |
| Excitatory neurons | *Slc17a7, Pcp4* |
| Inhibitory neurons | *Gad1, Synpr* |
| Oligodendrocytes | *Hapln2, Opalin, Ptgds* |
| Oligodendrocyte precursor cells (Opcs) | *Pdgfra, Olig2* |
| Astrocytes | *Slc1a3, Gja1, Aqp4, Phka1* |
| Microglia | *Cx3cr1, Csf1r, Dock8* |
| Pericytes | *Vtn* |
| Fibroblasts | *Nr4a2, Bnc2* |
| **Kidney Cell Type** | **Marker Gene(s)** |
| Pericytes | *Pdgfrb* |
| Endothelial | *Kdr, Ptprb* |
| Proximal Tubule (PT) | *Slc34a1*, *Slc13a3* |
| Proximal Straight Tubule (PST) | *Slc22a7*, *Atp11a* |
| Proximal Convoluted Tubule Segment 1 (PCTS1) | *Slc5a2, Slc5a12* |
| Proximal Convoluted Tubule Segment 2 (PCTS2) | *Fxyd2* |
| Loop of Henle (LOH) | *Slc12a1* |
| Collecting Duct Principal cells (CDPC) | *Aqp2, Hsd11b2* |
| Distal Convoluted Tubule (DCT) | *Slc12a3* |
| Macrophages | *Runx1, Ptprc* |
| Distal Loop of Henle (DLH) | *Bst1, Akr1b3* |
| Collecting Duct Intercalated cells (CDIC) | *Atp6v1g3, Atp6v0d2* |
| CDIC type A | *Aqp6* |
| CDIC type B | *Hmx2* |
| B cells | *Cd79b, Bank1* |
| Podocytes | *Nphs1, Nphs2, Wt1* |
| Fibroblasts | *Pdgfra* |
| Smooth muscle cells | *Atp1a2* |

**Figure S3: Marker genes for cortex and kidney cell type annotation and proportion of nuclei**


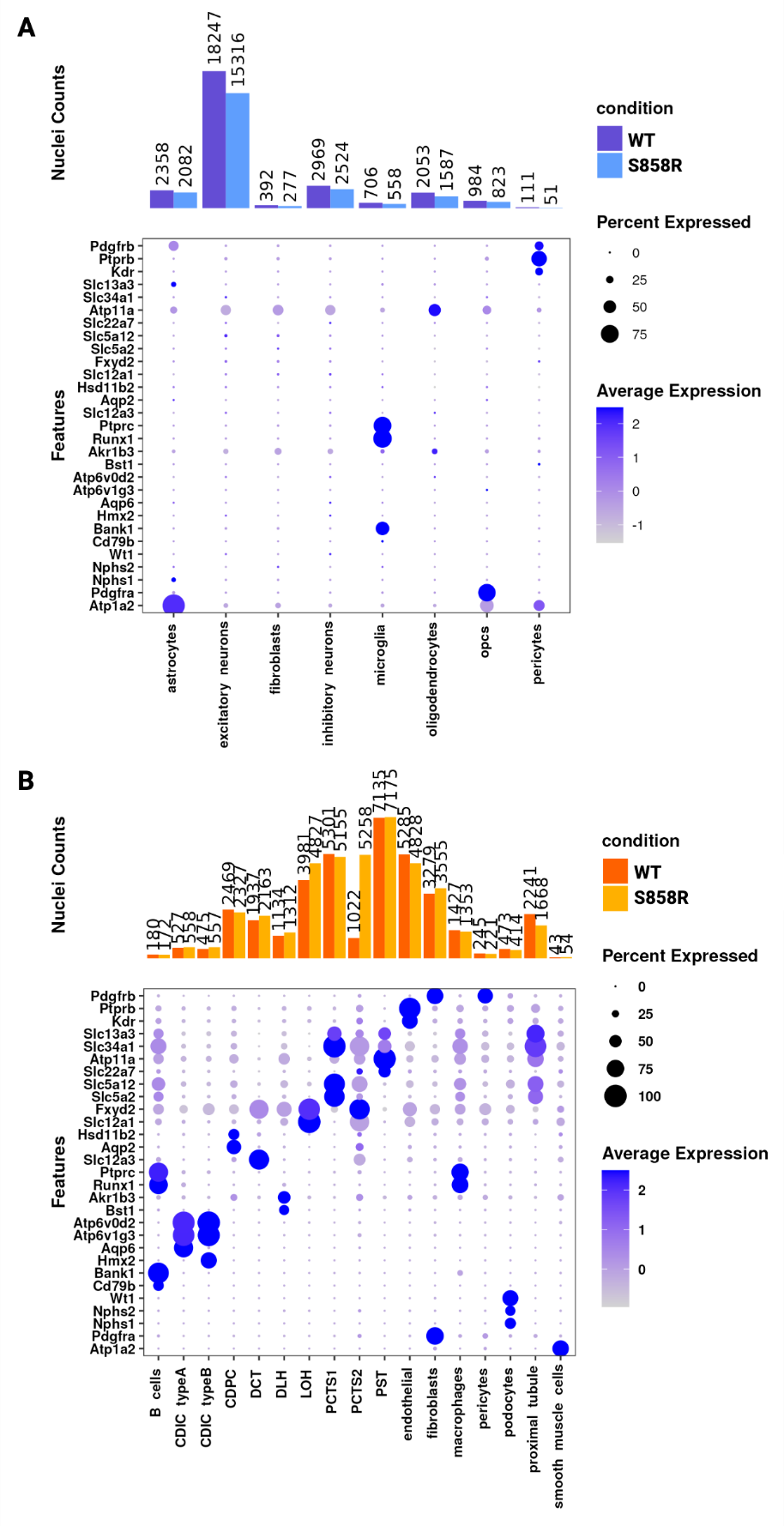


**Figure S4: UMAP of Setbp1 S858R snRNA-seq by condition and sample**


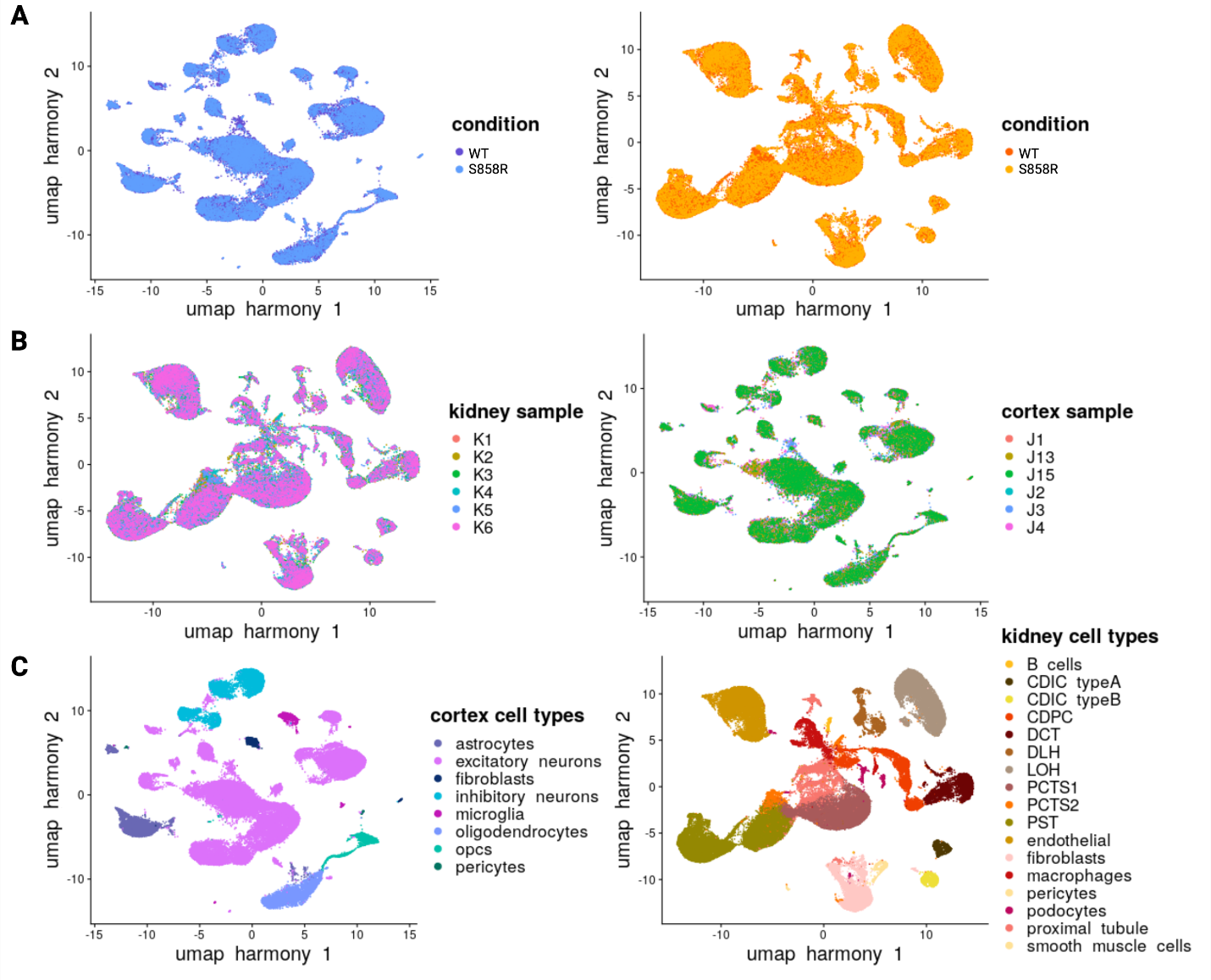


**Figure S5: gprofiler2 GO pathway analysis on predicted Setbp1 target genes upregulated in S858R excitatory neurons**
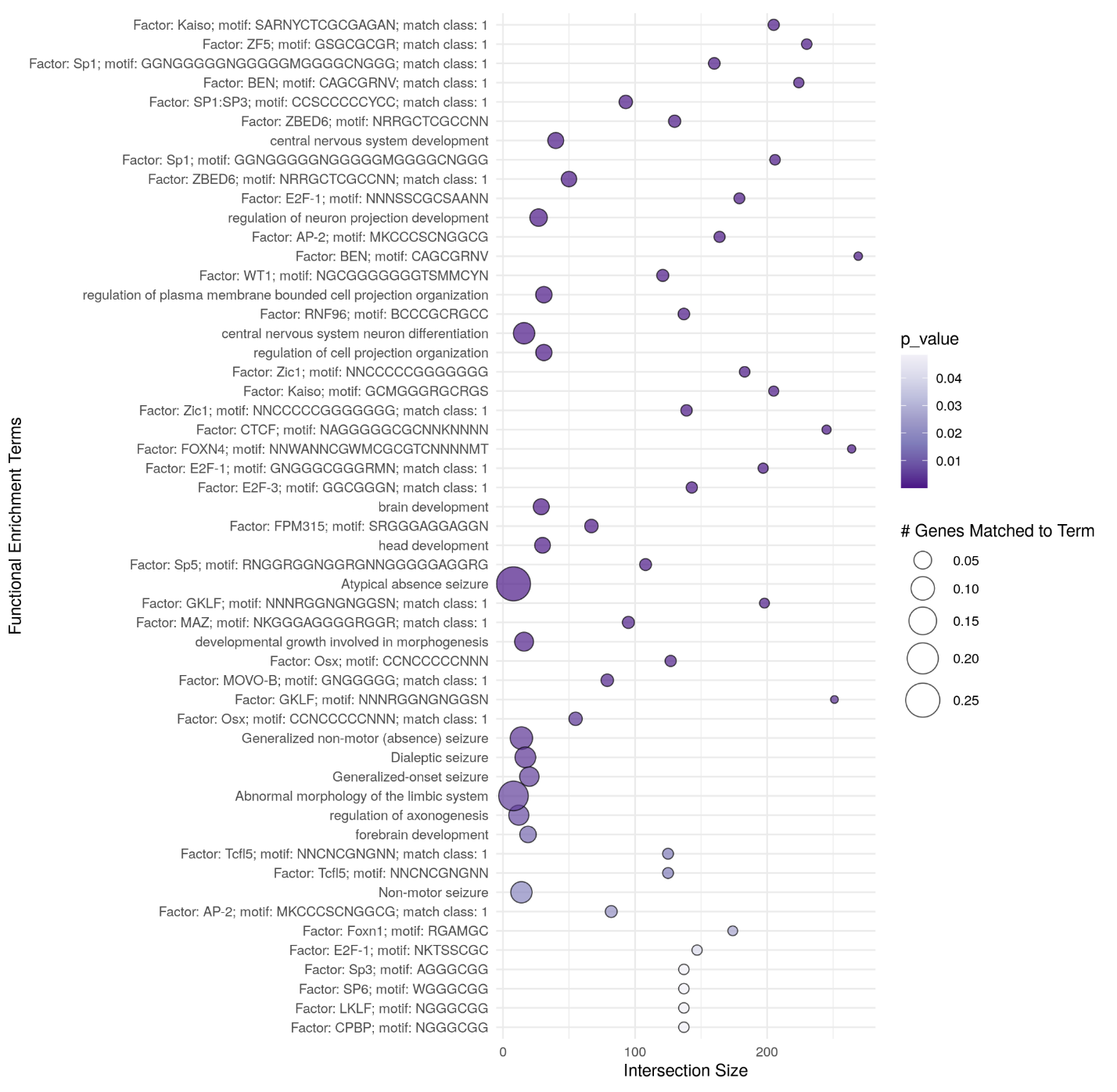


**Figure S6: gprofiler2 GO pathway analysis on predicted Setbp1 target genes downregulated in S858R excitatory neurons**
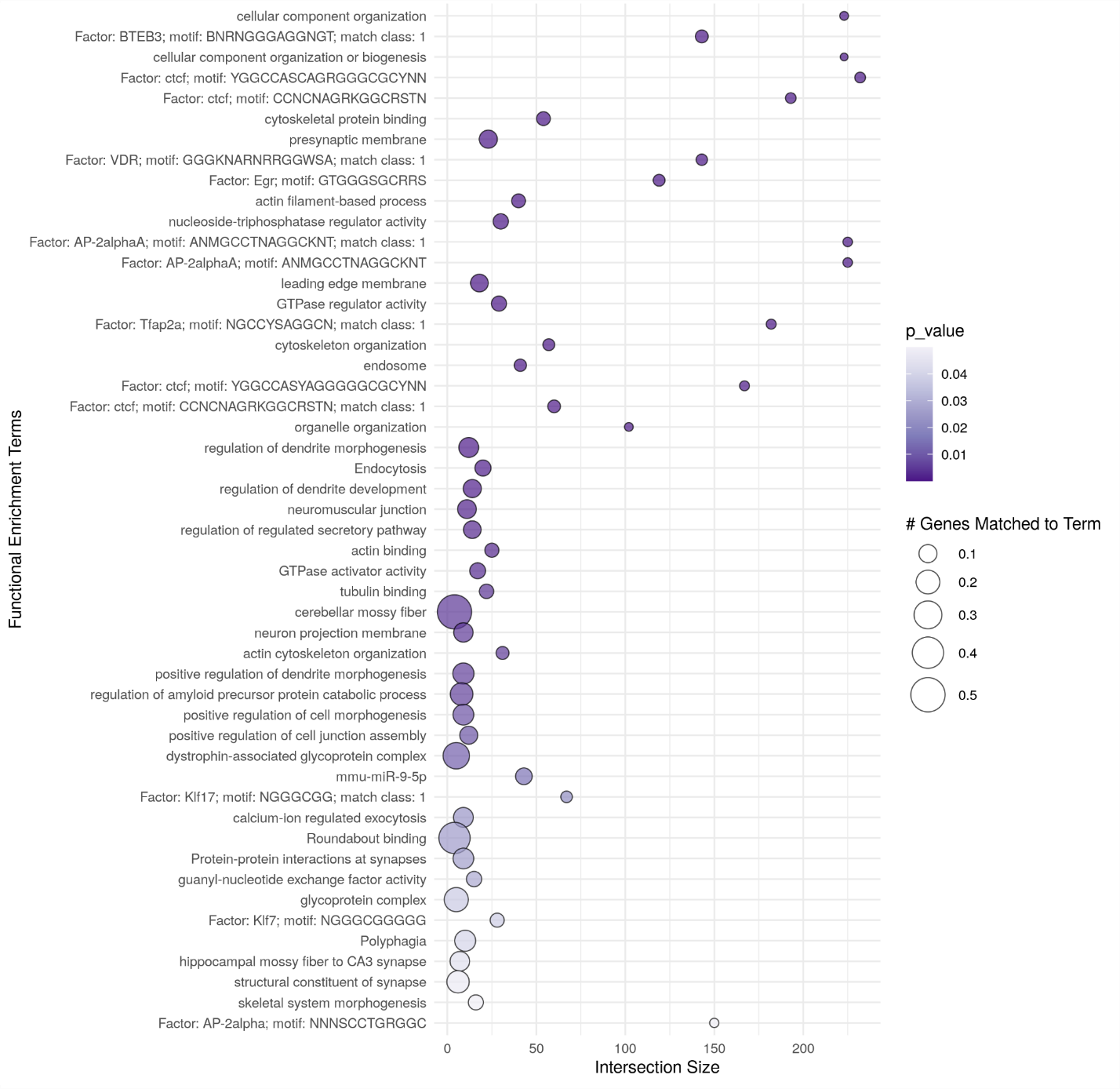


**Figure S7: gprofiler2 GO pathway analysis on predicted Setbp1 target genes downregulated in S858R astrocytes**


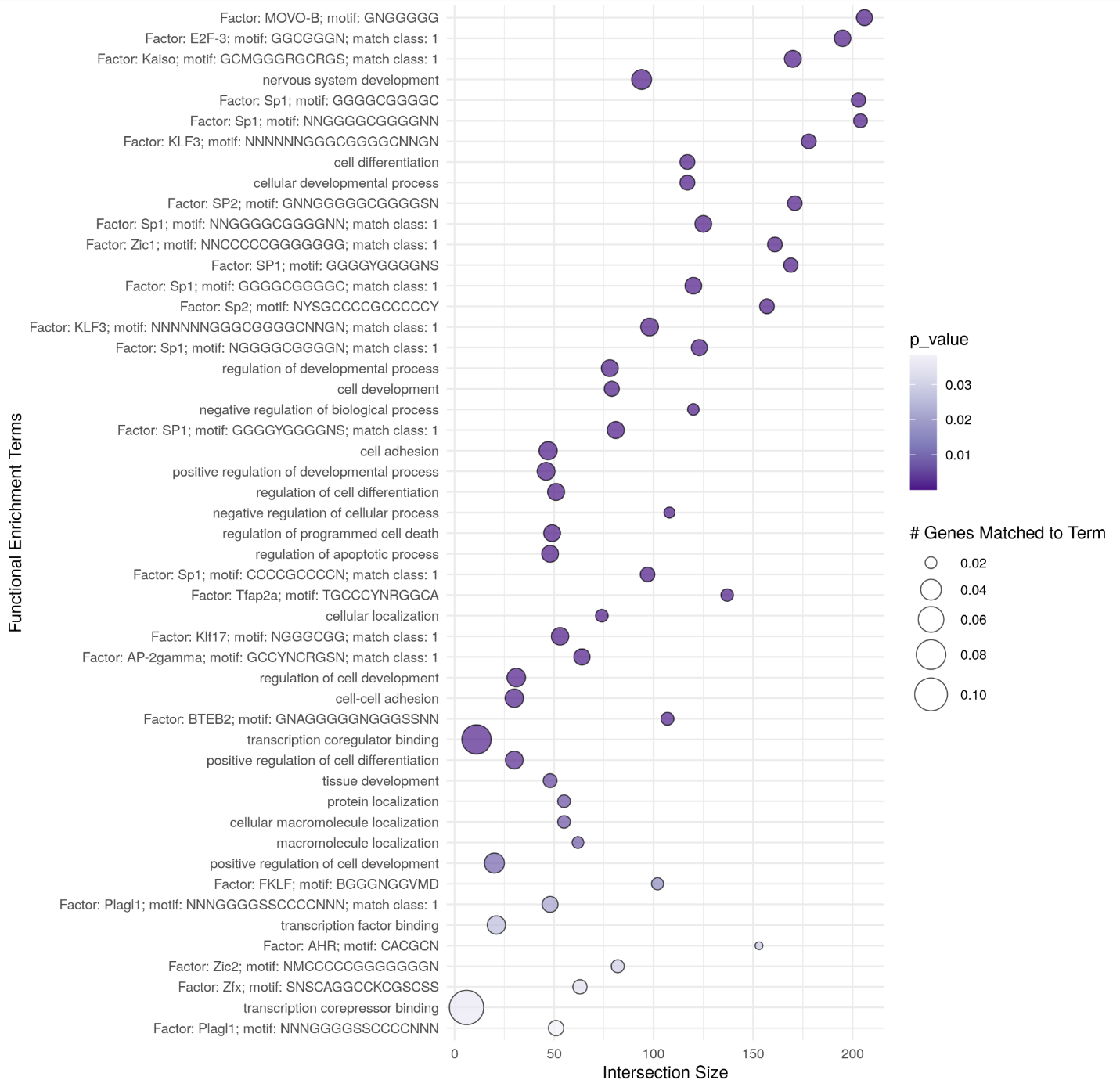


**Figure S8: gprofiler2 GO pathway analysis on predicted Setbp1 target genes downregulated in S858R inhibitory neurons**


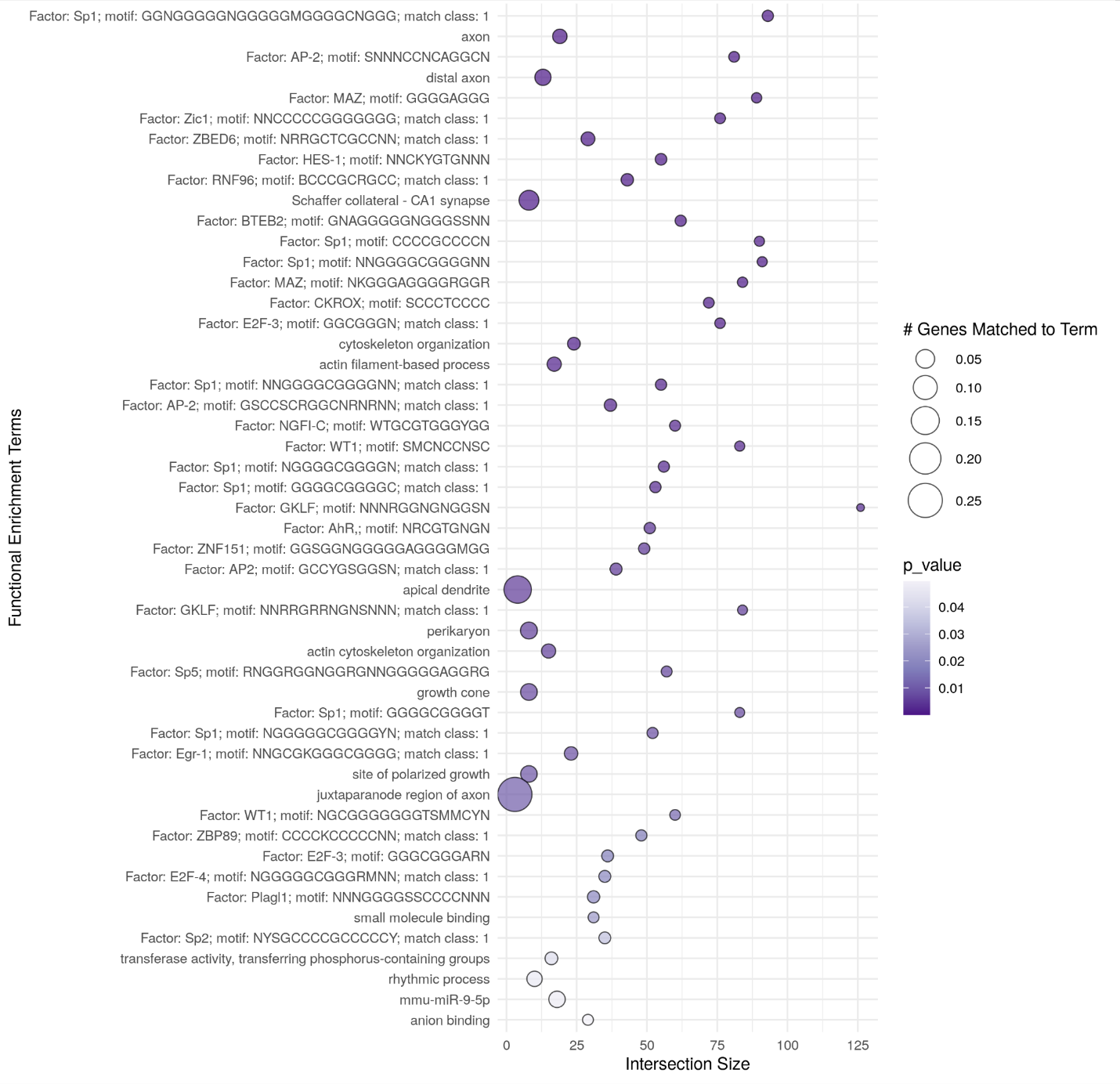


**Figure S9: gprofiler2 GO pathway analysis on predicted Setbp1 target genes downregulated in S858R microglia**


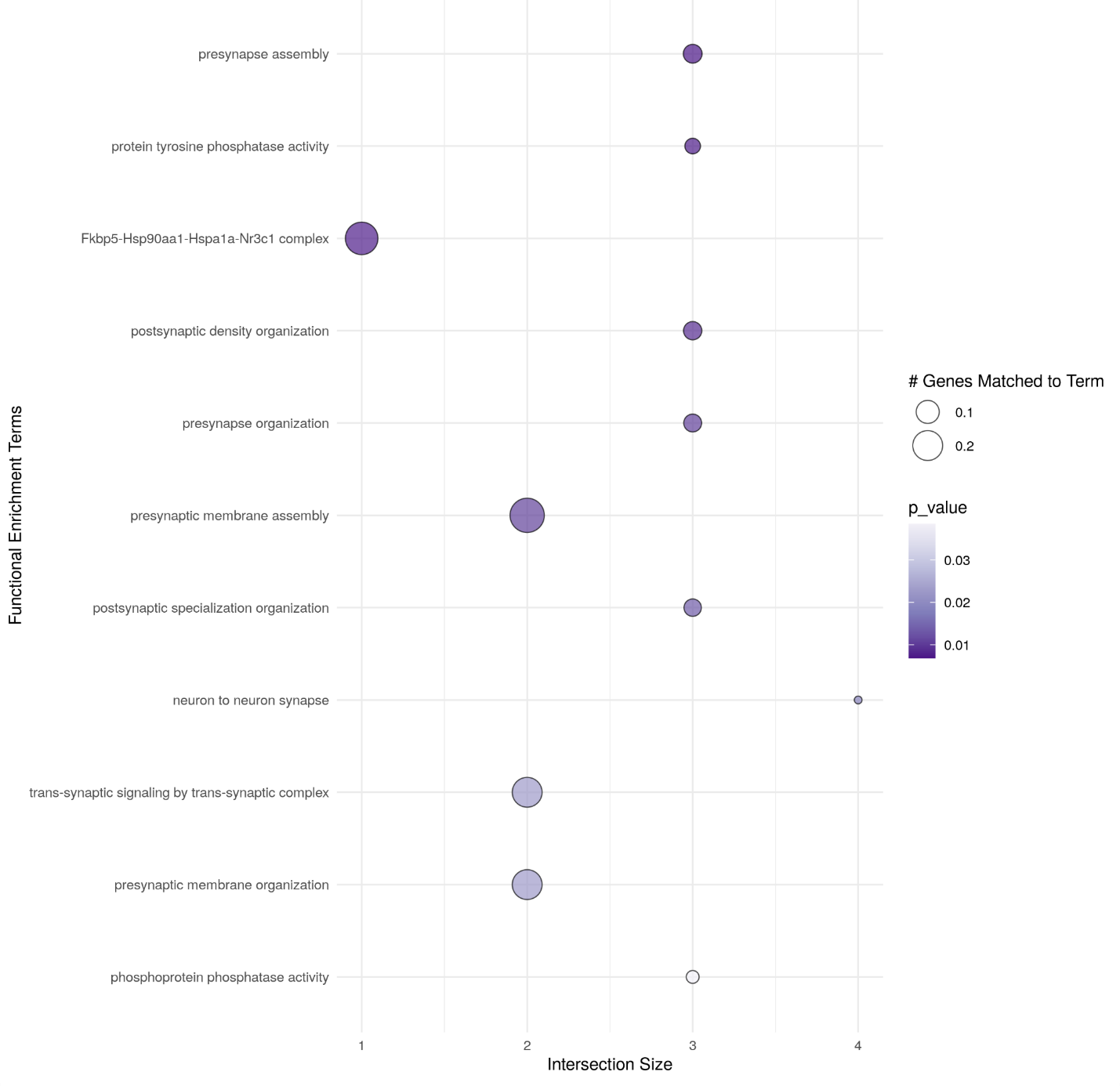


**Figure S10: gprofiler2 GO pathway analysis on predicted Setbp1 target genes upregulated in S858R microglia**
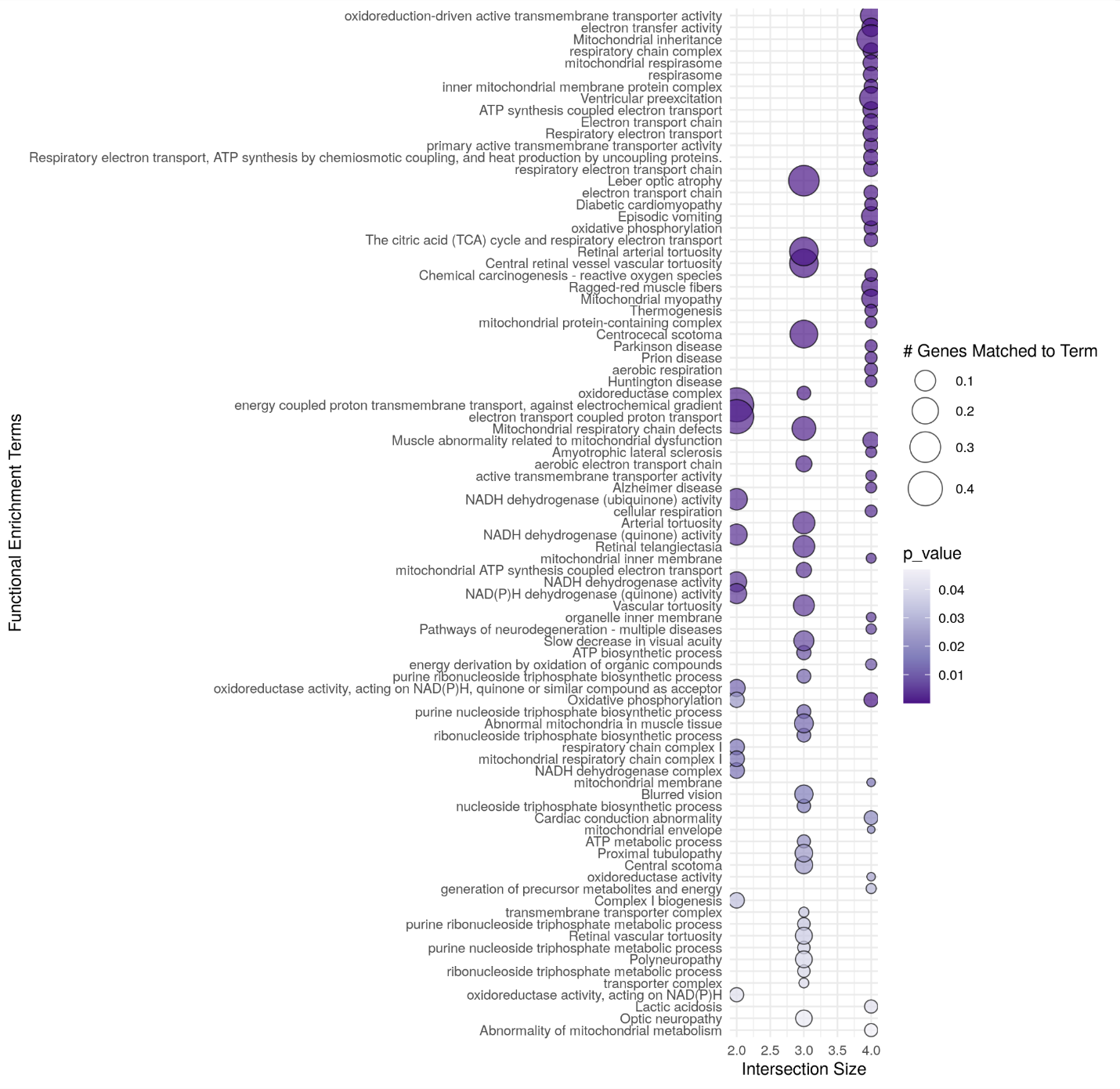


**Figure S11: S858R kidney cystic kidney signature**


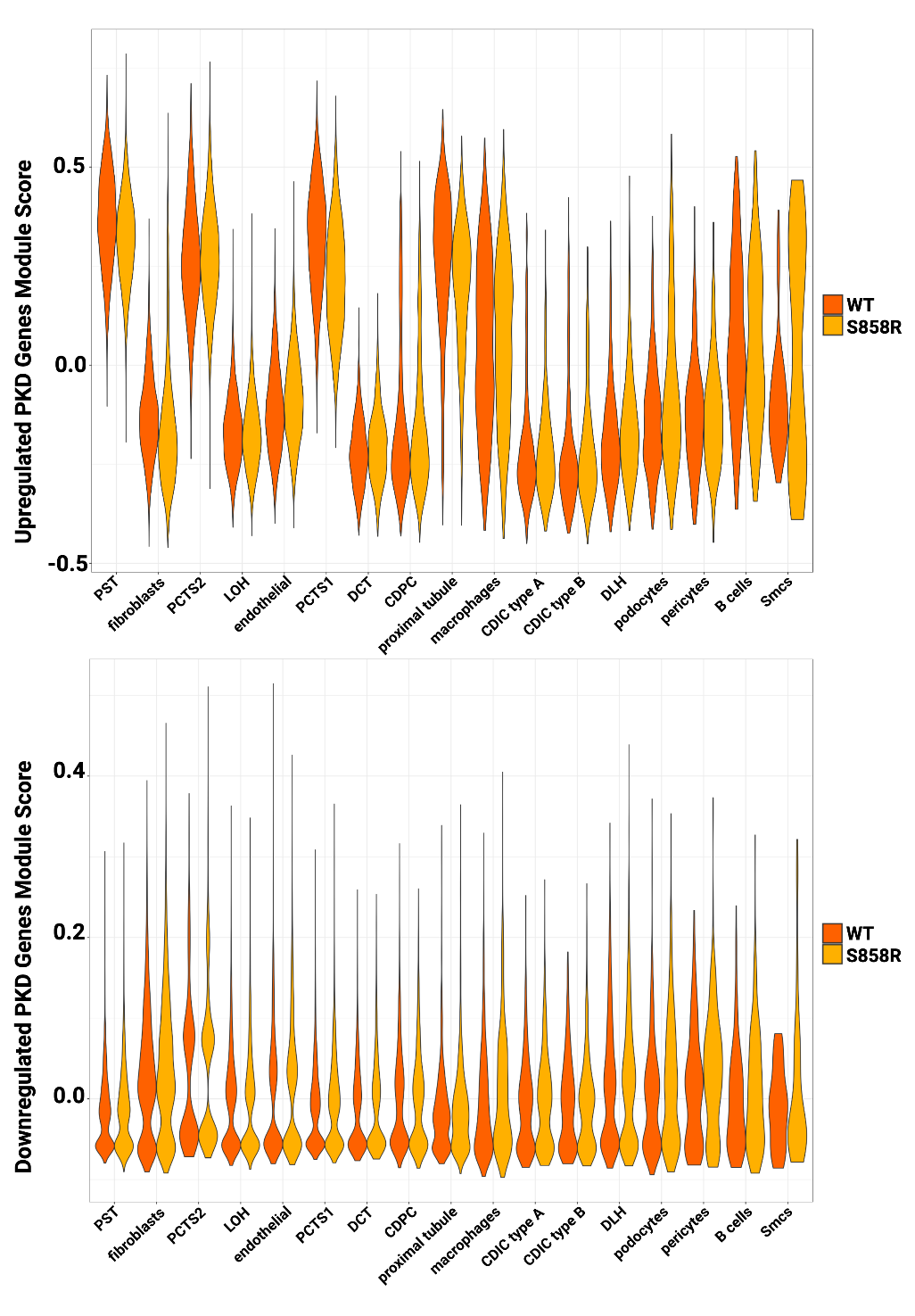
\

**Figure S12: S858R kidney failed repair signature**
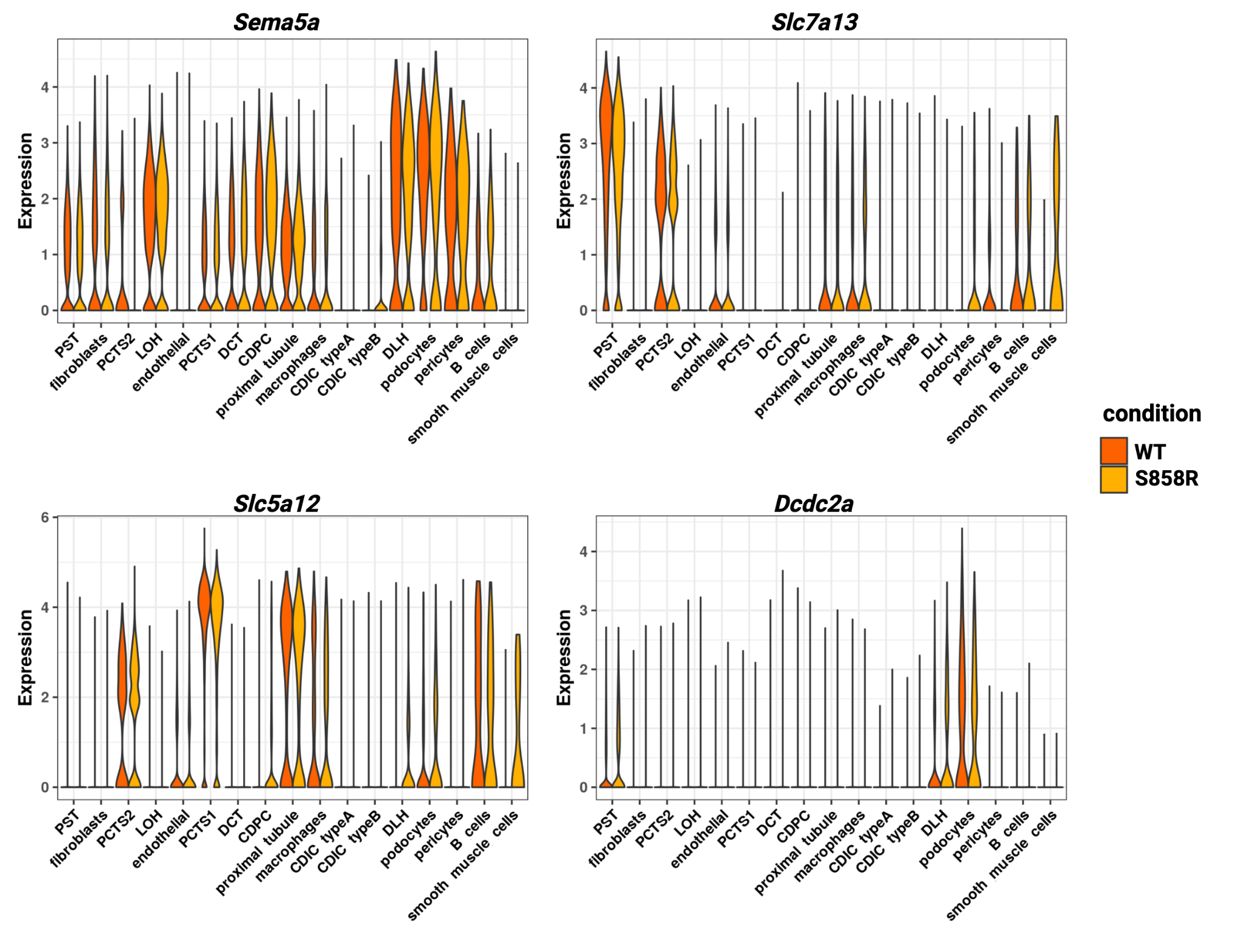


**Figure S13: SGS specific Hallmark pathways for cerebral cortex and all hallmarks for kidney with VISION analysis**

######
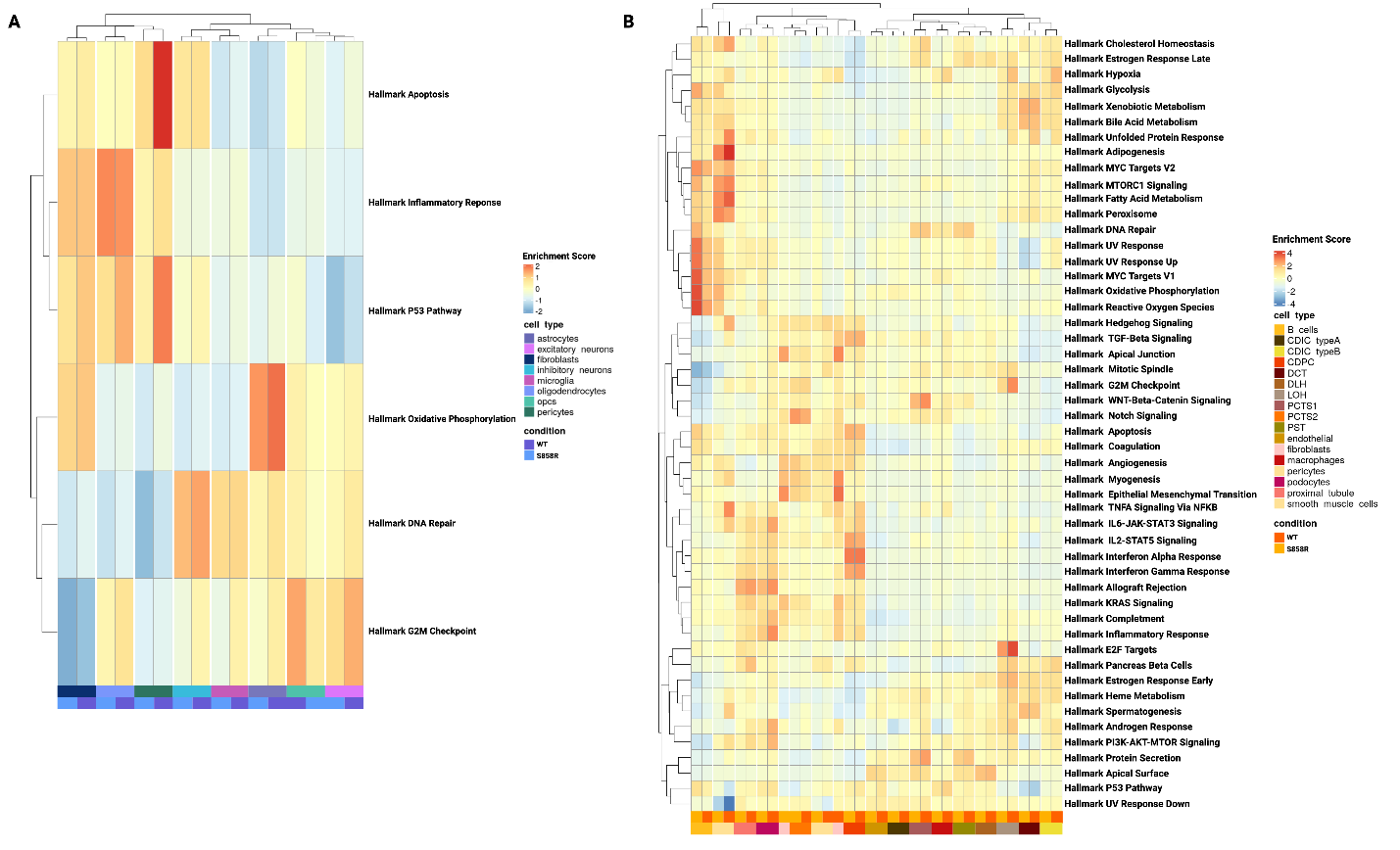


**Figure S14: Expression of reactive astrocyte marker Gfap in cerebral cortex**


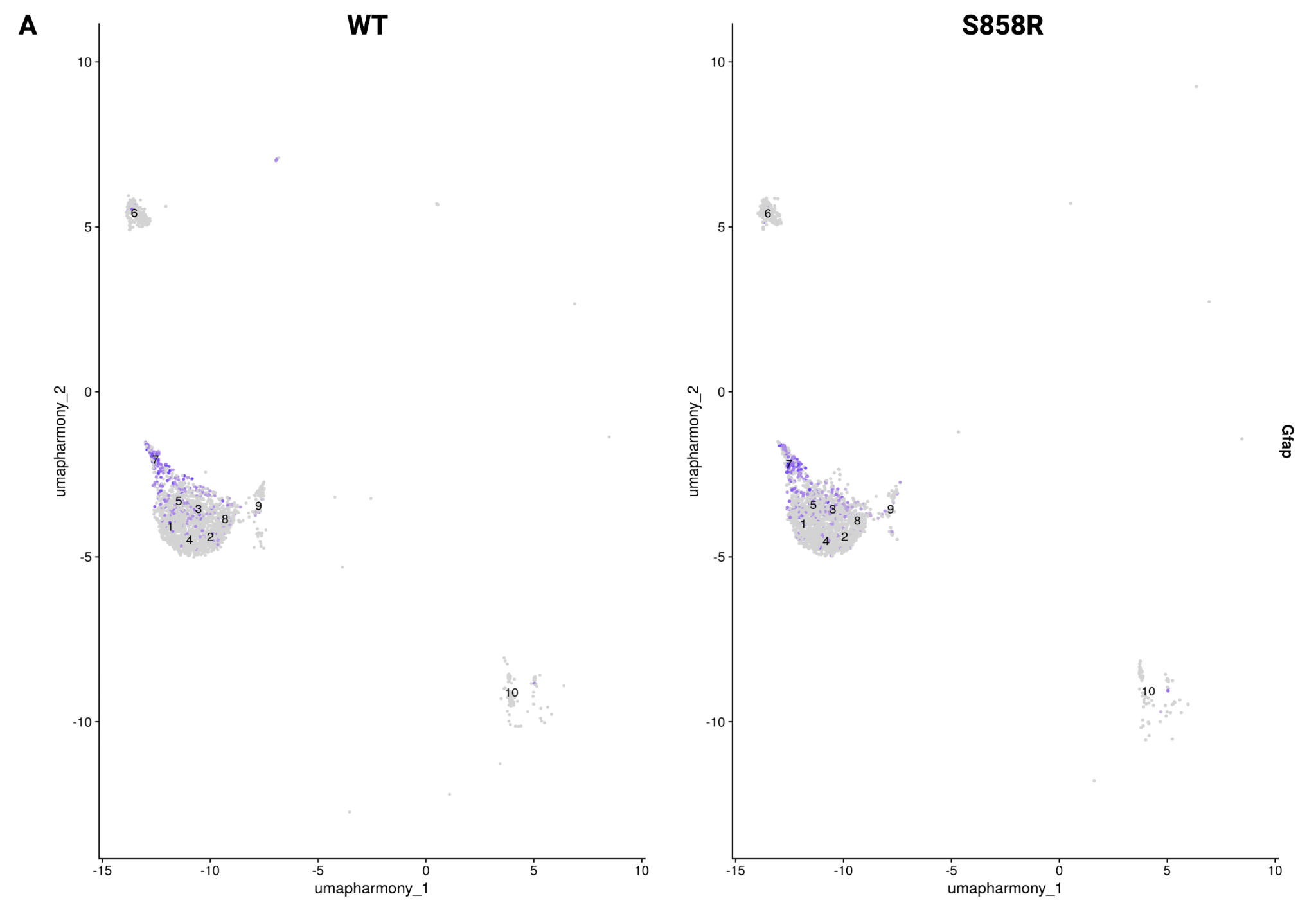


######

**Figure S15: Set has similar, ubiquitous expression across all cell types in both tissues**


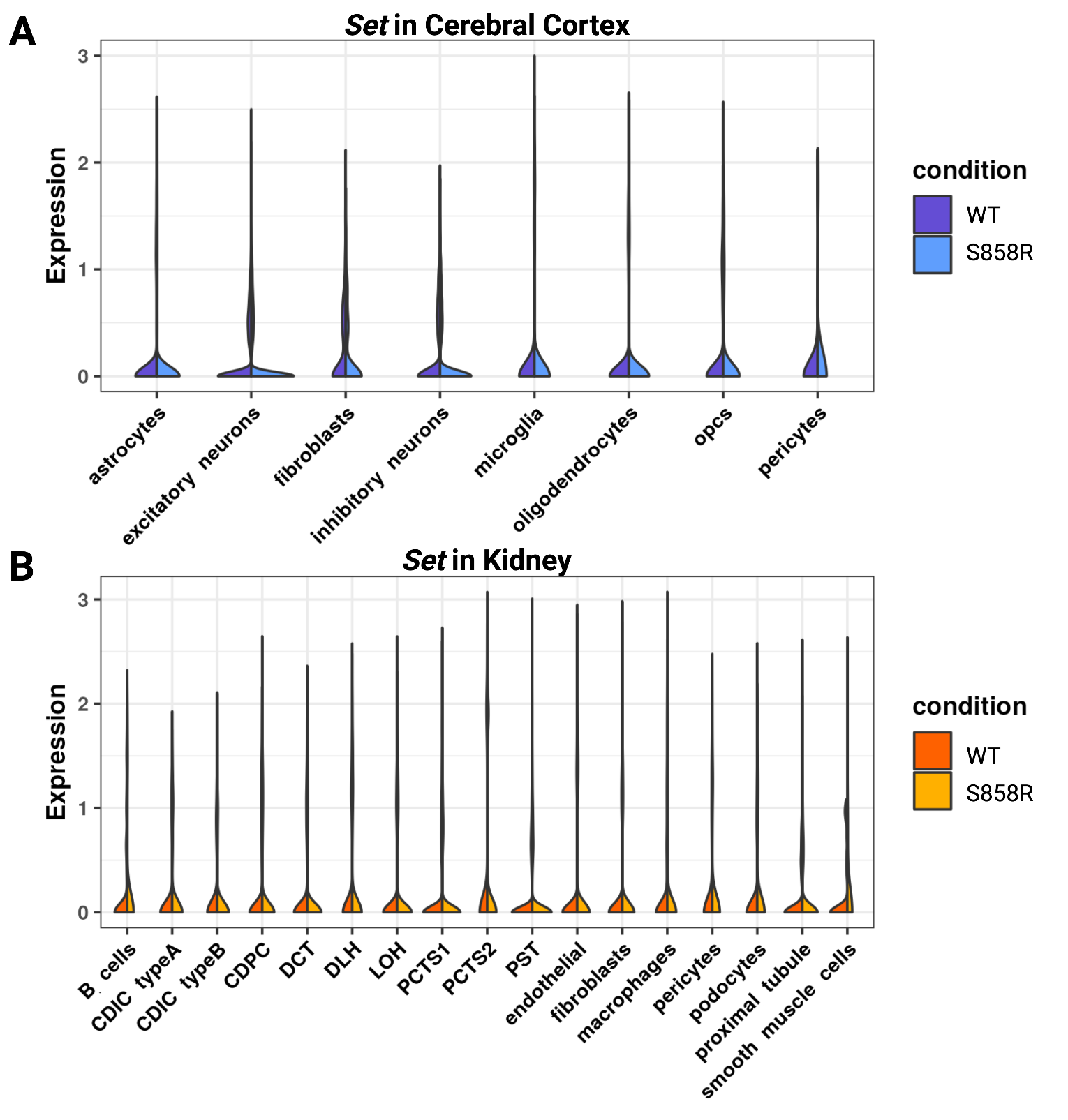


**Figure S16: Ppp2ca decrease in expression of S858R astrocytes (almost lack of) compared to WT whereas the inverse was observed in microglia and has similar expression across all cell types in kidney**


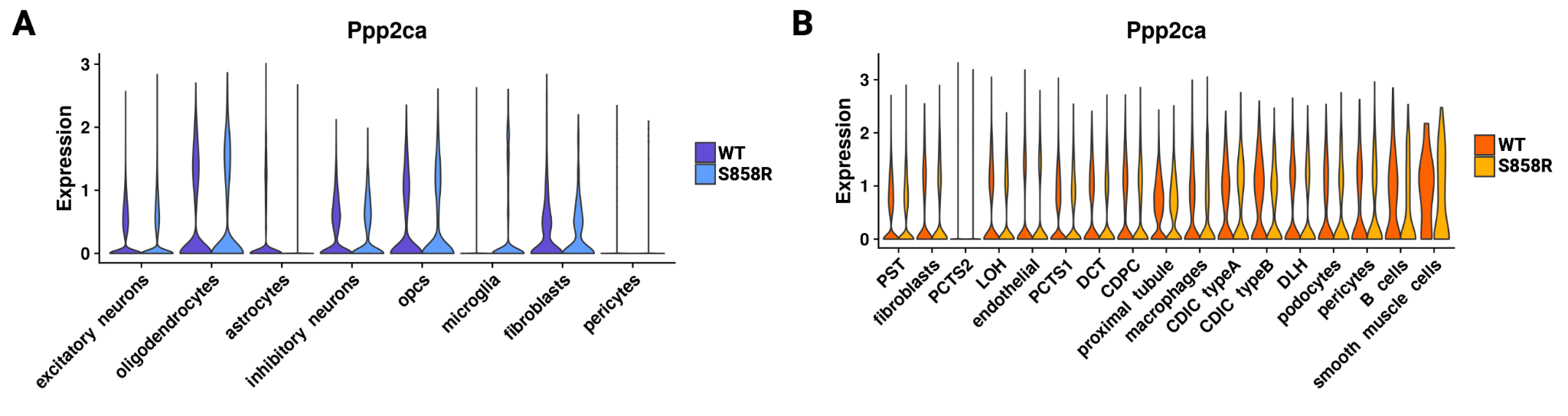


**Figure S17: All Hallmark pathways for cerebral cortex with VISION analysis**


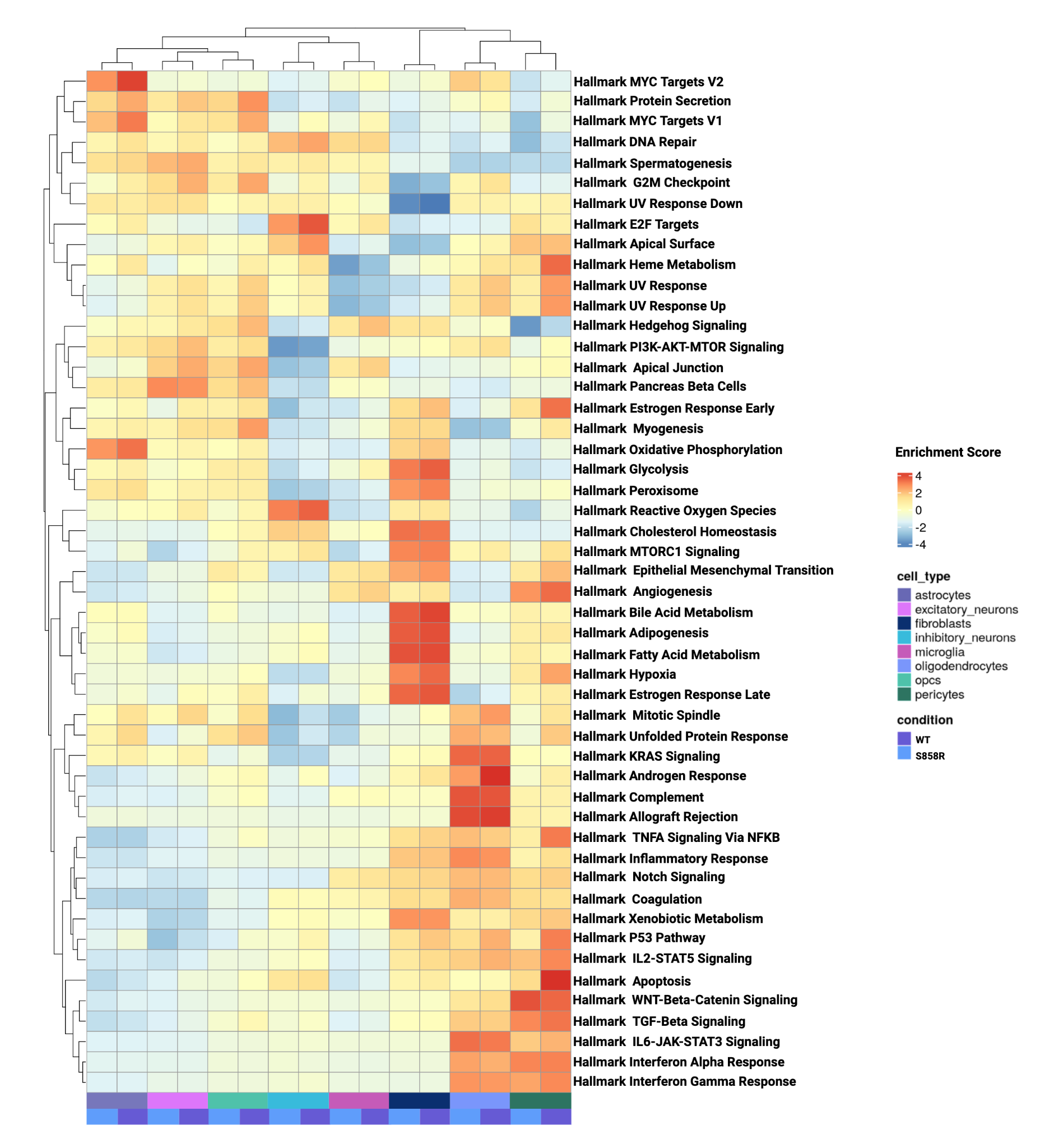


**Figure S18: Control Cooperativity and Regulation**
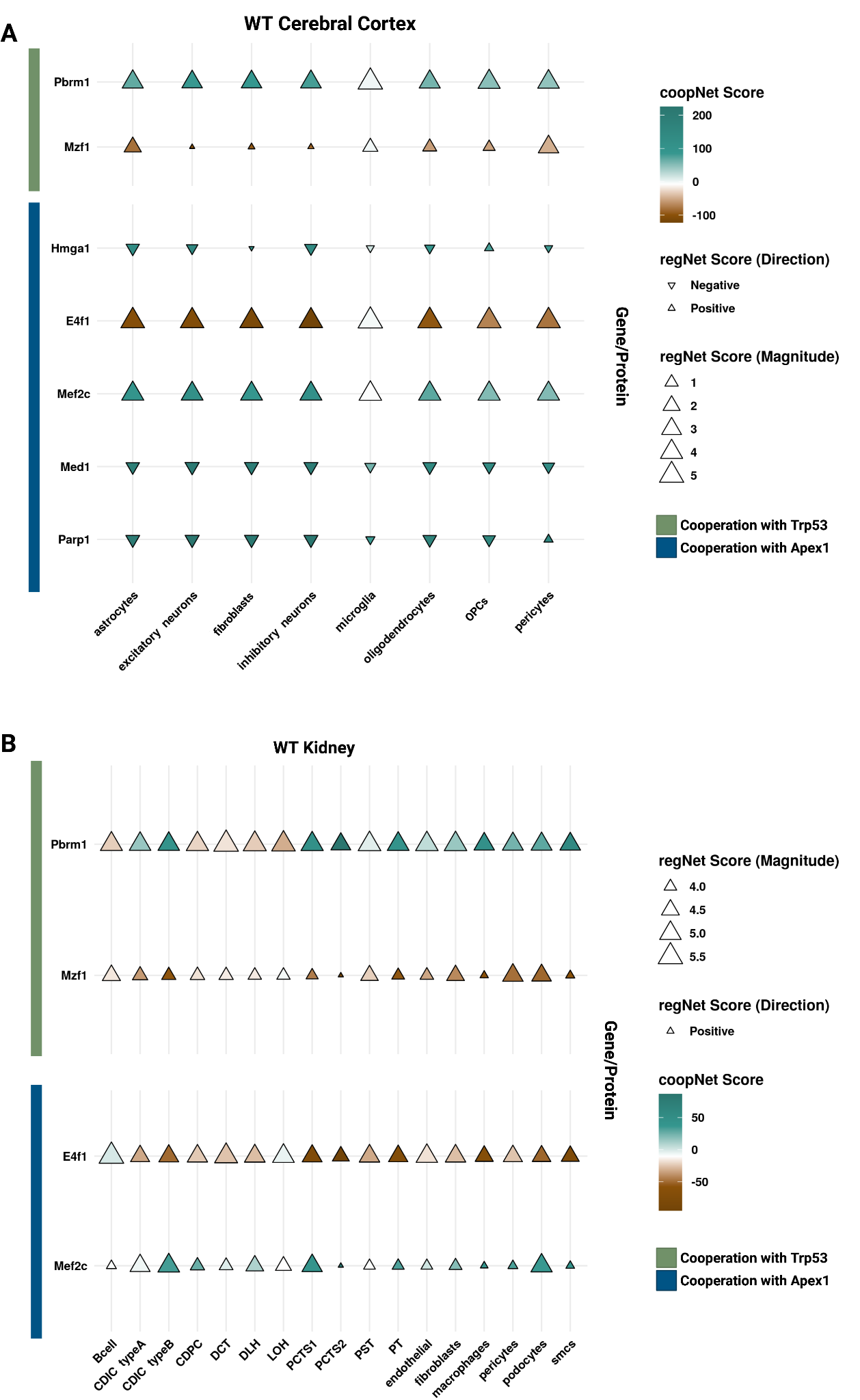


**Table S8: STRINGdb prior evidence for proteins of interest**

| **STRING score** | **Protein 1** | **Protein 2** |
| --- | --- | --- |
| 873 | Parp1 | Apex1 |
| 953 | Parp1 | Trp53 |
| 987 | E4f1 | Trp53 |
| 654 | Med1 | Trp53 |
| 207 | Apex1 | E4f1 |
| 735 | Trp53 | Pbrm1 |
| 287 | Apex1 | Hmga1 |
| 717 | Trp53 | Hmga1 |
| 396 | Trp53 | Mef2c |
| 537 | Trp53 | Mzf1 |
